# Attenuation and Immunogenicity of a Live High Pathogenic PRRSV Vaccine Candidate with a 32-Amino Acid Deletion in the nsp2 Protein

**DOI:** 10.1155/2014/810523

**Published:** 2014-06-09

**Authors:** Wenhui Lu, Baoli Sun, Jianyue Mo, Xiduo Zeng, Guanqun Zhang, Lianxiang Wang, Qingfeng Zhou, Ling Zhu, Zhili Li, Qingmei Xie, Yinzuo Bi, Jingyun Ma

**Affiliations:** ^1^College of Animal Science, South China Agricultural University, Tianhe District, Wushan Road, Guangzhou, Guangdong 510642, China; ^2^Guangdong Wen's Foodstuff Group Co., Ltd., Yanjiang Street, Xinxing, Guangdong 527400, China; ^3^College of Veterinary Medicine, South China Agricultural University, Tianhe District, Wushan Road, Guangzhou, Guangdong 510642, China

## Abstract

A porcine reproductive and respiratory syndrome virus (PRRSV) QY1 was serially passed on Marc-145 cells. Virulence of different intermediate derivatives of QY1 (P5, P60, P80, and P100) were determined. The study found that QY1 had been gradually attenuated during the in vitro process. Pathogenicity study showed that pigs inoculated with QY1 P100 and P80 did not develop any significant PRRS clinic symptoms. However, mild-to-moderate clinical signs and acute HP-PRRSV symptoms of infection were observed in pigs inoculated with QY1 P60 and P5, respectively. Furthermore, we determined the whole genome sequences of these four intermediate viruses. The results showed that after 100 passages, compared to QY1 P5, a total of 32 amino acid mutations were found. Moreover, there were one nucleotide deletion and a unique 34-amino acid deletion found at 5′UTR and in nsp2 gene during the attenuation process, respectively. Such deletions were genetically stable in vivo. Following PRRSV experimental challenge, pigs inoculated with a single dose of QY1 P100 developed no significant clinic symptoms and well tolerated lethal challenge, while QY1 P80 group still developed mild fever in the clinic trial after challenge. Thus, we concluded that QY1 P100 was a promising and highly attenuated PRRSV vaccine candidate.

## 1. Introduction

Porcine reproductive and respiratory syndrome (PRRS) is a severe viral disease in pigs, characterized by reproductive failure in sows and respiratory problems in pigs. Since its emergence in the US in the 1980s, PRRS was found worldwide and had caused great financial losses to the swine industry in the world [1–4]. PRRS is caused by PRRS virus (PRRSV), which belongs to order Nidovirales, family Arteriviridae, genus* Arterivirus* [5]. PRRSV is susceptible to genetic diversity. On the basis of phylogenetic analysis of PRRSV isolates, the virus can be divided into two genotypes: type I (Europe-like) typified by LV and type II (NA-like) typified by VR-2332 [6, 7]. Within the type II PRRSV, it is overall divided into 9 monophyletic lineages [8]. Both type I [9] and type II PRRSV were reported in China. It was noticed that the variant PRRSV which was also named highly pathogenic PRRSV (HP-PRRSV), emerged in 2006, had affected more than 200 millions pigs, and had caused huge economic losses to Chinese swine industry [3].

PRRSV has a positive-sense RNA genome of approximately 15.1–15.5 kb. The genome contains at least 10 open reading frames (ORFs) [10]. The ORF1a and ORF1b are located downstream of 5′ untranslated region (UTR) and occupy around two-thirds of the genome and yield at least 14 smaller viral nonstructural proteins (NSPs, Nsp1*α*, Nsp1*β*, and Nsp2-12) related to the viral transcription and replication. ORF2a, ORF2b, and ORFs 3–7, located upstream of the 3′UTR, encode the minor structural proteins (GP2, E, GP3, and GP4) and major structural proteins (GP5, M, and N), respectively [11–15]. An additional novel structural protein encoded by ORF5a was identified to be important for PRRSV replication [10, 16].

Live attenuated virus vaccine is considered to be the most economic method to achieve immunization [17]. Several different live-attenuated vaccines had been developed to control this disease and these vaccination strategies had greatly reduced the economic loss caused by PRRS [18–21]. Live-attenuated vaccines were somehow an effective way of inducing immunity and protecting herds from losses associated with infections by highly virulent strains of PRRSV [22]. All of the commercially available live-attenuated vaccines were derived from field wild-strains after sequential passages of the virus on cell line; it was shown that both structural and nonstructural viral proteins and perhaps the interaction of different proteins led to PRRSV virus attenuation in vivo [18, 19, 23–25] and overattenuation of HP-PRRSV would result in insufficient immunogenicity, which had been reported [26]. Thus, a satisfactory balance between attenuation and good immunogenicity for PRRSV live vaccine requires a better understanding on the PRRSV attenuation. In the present study, we described a PRRSV isolate QY1 which was serially passed on Marc-145 cells; genome sequences and the level of attenuation of different derivative of QY1 were determined as well. Additional studies were carried out to evaluate the immunogenicity of its attenuated phenotype for a better understanding of the possible relationship between genetic mutations and PRRSV attenuation.

## 2. Materials and Methods

### 2.1. Virus Culture

PRRSV strain QY1 was isolated in June 2007. Marc-145 cell culture was applied to virus propagation, and the inoculated monolayers of Marc-145 cells were placed at 37°C, 5% CO_2_ with Dulbecco's modified Eagle's medium (DMEM) containing 2% fetal bovine serum (Gibco) when 70%–80% cytopathic effects (CPE) was visible. The cell culture supernatants were harvested by freezing and thawing for 3 times, which were diluted as the ratio of 1 : 50 or 1 : 100 to start the next passage. Virus purification and titer assessment were carried out every 10 passages.

### 2.2. Virus RNA Extraction and Genome Sequencing

Total viral RNAs were extracted from different QY1 virus passages using TRIzoL Reagent (Invitrogen, USA). Complete genomic sequences of P5, P60, P80, and P100 were determined, and the ORF5 gene was determined at every 10-passage interval as a viral mutation insight. Total RNA was dissolved in nuclease-free water and stored at −70°C for further use. The reverse transcription (RT) and the polymerase chain reaction (PCR) were conducted using PrimeScript One Step RT-PCR kit (TaKaRa, Japan). A 3′-full RACE Kit (TaKaRa, Japan) was employed to amplify the 3′UTR according to instructions of the manufacturer. 17 primers pairs were designed to amply the full length of viral-genome based on sequence of the VR-2332 [27]. PCR products were purified from agarose gel by using an E.Z.N.A. Gel Extraction Kit (OMEGA, USA) according to the manufacture's recommendation. The PCR products were cloned into pMD19-T vector (TaKaRa, Japan) and sent to Shanghai Sango Biotech, China, for sequencing. A quality sequence represented at least threefold genome coverage and the sequence results were analyzed using DNAStar lasergene version 7.2.

### 2.3. Pathogenicity Study Design and Experimental Challenge

Institutional and national guidelines for the care and use of animals were followed and all experimental procedures involving animals were approved by the Committee of Animal Experiments of South China Agricultural University (approval ID 201004152). All efforts were made to minimise suffering.

A total of fifty 35-day-old PRRSV-free piglets were obtained from a farm that was negative for PRRSV and PCV2 infections. The piglets were randomly divided into five groups with 10 animals in each group. Each group of piglets was housed separate from other groups in biological safety level 2 (BSL2) facilities provided by Guangdong Dahuanong Animal Health Products Co., Ltd. At 6 weeks of age, piglets in groups 1, 2, 3, and 4 were inoculated intramuscularly with 2 × 10^5^ tissue culture infective does (TCID50) QY1 virus of P5, P60, P80, and P100, respectively. Group 5 was intramuscularly injected with Dulbecco's Modified Eagle's Medium (DMEM), which was used as the negative control. Then piglets were monitored daily for clinical signs, including anorexia, lethargy, diarrhea, dyspnoea, and body temperature. Animals were weighed at 0, 7, 14, 21, and 28 days after inoculation (dpi). Serum samples were collected on 0, 3, 7, 10, 14, 21, and 28 dpi. Five pigs from each group were randomly selected and were necropsied on 14 dpi, followed by lungs examination for gross and microscopic changes.

The remaining pigs (*n* = 5) of groups 3, 4, and 5 were intramuscularly injected with 2 × 10^5^ of the P5 virus on 28 dpi for experimental challenge, respectively. Animals were monitored daily for the presence of clinical signs of anorexia, lethargy, diarrhea, and dyspnoea and body temperature. Blood samples were collected on 28, 35, and 42 dpi. All pigs were necropsied on the 14th day after challenge, and gross pathological lung lesions were evaluated. Lung tissues were collected for histological examination as well.

### 2.4. Serology

Serum samples collected on 0, 7, 14, and 21 dpi were used for PRRSV specific antibody responses using a commercial ELISA kit 2XR (IDEXX Laboratories Inc., Westbrook, ME) according to the manufacturer's instructions. Samples with sample-to-positive (S/P) ratios ≥0.4 were considered positive for antibodies against PRRSV. In addition, serum samples from 21, 28, 35, and 42 dpi were used for virus neutralization assays as described by Plagemann et al. [28].

### 2.5. Viremia

Virus isolation and viral titration assay in serum were conducted. Briefly, 50 *μ*L of serum was added on a monolayer of Marc-145 cells in 1 well of a 24-well plate for virus isolation. Each well was examined for cytopathic effect and assessed as positive or negative daily for one week of culture. Titration was performed by preparing 10-fold dilutions of each positive sample and adding 50 *μ*L of each dilution to 4 wells of a monolayer of Marc-145 cells in a 96-well plate. The 50% tissue culture infective dose (TCID_50_) per mL was calculated according to the method of Reed and Muench.

### 2.6. Histological Examination

Samples of lung (two sections from the cranial lobe and one from each of middle, accessory, and caudal lung lobes) were collected and fixed in 10% neutral buffered formalin and routinely processed for histopathological examination. The microscopic sections were examined in blind fashion and assigned a score for severity of interstitial pneumonia (0 to 4) as previously described [29].

### 2.7. Statistical Analysis

Statistical analysis of antibody and virus titers was performed using SPASS 7.0 software. One-way analysis of variance (ANOVA) was used to evaluate the differences among the geometric mean antibody and virus titers. The level of statistically significant difference was set as *P* < 0.05.

## 3. Result

### 3.1. Clinical Signs and Weight Gain

**Figure 1:**
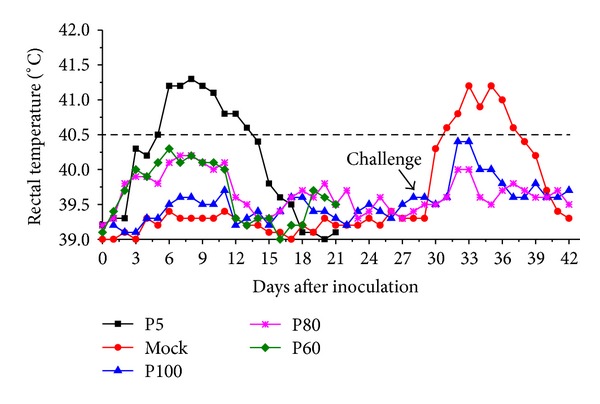
Mean rectal temperature following inoculation with different QY1 passage virus. 28 days after inoculation, five pigs in P80, P100, and mock groups were challenged with QY1 P5.

**Figure 2:**
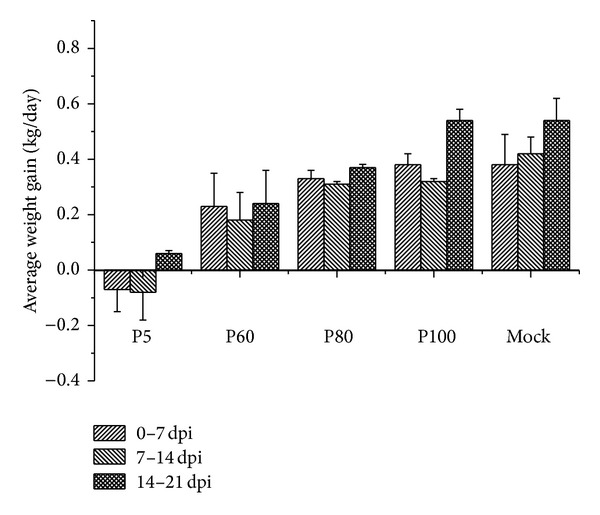
Average weight gains after PRRSV inoculate (0–21 dpi). Data were express as mean ± S.D. of the numbers of pigs alive at the time of the measurement.

The negative-control group showed no clinical symptoms and was PRRSV free until the end of the pathogenicity study (up to 28 dpi). All piglets infected with QY1 P5 virus developed typical clinical symptoms of HP-PRRSV including high fever, anorexia, depression, lethargy, dyspnea, and skin cyanosis and 2/10 piglets in this group died on 9 dpi and 11 dpi, respectively. Other pigs of this group began to decrease in severity on 12 dpi except that two pigs still showed severe weakness and moribund condition and were euthanized on 14 dpi. Febrile response was shown in Figure 1. All pigs inoculated with P5 exhibited high fever (≥40.5°C) on 4 dpi, which lasted for 6 days. Pigs inoculated with QY1 P60 exhibited mild to moderate clinical symptoms, such as anorexia, depression, and lethargy and four pigs in this group showed moderate dyspnea and high fever but the average body temperature of this group was below 40.5°C. The P60 group exhibited clinical symptoms beginning on 3 or 4 dpi, which reached peak on 10 dpi and resolved from 14 to 21 dpi. 4/10 pigs infected with QY1 P80 exhibited moderate fever (40°C–40.5°C) on 5 dpi, which lasted for 5 days. Inoculation with P80 clearly induced elevated temperature following inoculation, suggesting that there was residual virulence in the viruses. In contrast, animals inoculated with QY1 P100 did not show any significant clinical symptoms throughout the experiment. Rectal temperature of pigs infected with QY1 P100 was within normal range; the performance on weight gaining was shown in Figure 2. No significant difference in weight gaining was observed among P80, P100, and control groups. The least weight gain was found among pigs infected with the virulent virus QY1 P5 followed by the moderate virulent virus QY1 P60.

At 28 dpi, pigs inoculated with either QY1 P80 (*n* = 5) or P100 (*n* = 5) were challenged with QY1 P5. Pigs in these two groups did not show high fever or clinical symptoms after challenge exposure, which indicated that these pigs were completely protected following inoculation with either QY1 P80 or P100. After challenge exposure, all pigs in group 5 developed acute clinical symptoms of HP-PRRSV, including anorexia, lethargy, diarrhea, dyspnoea, and persistent high body temperature. Most importantly, 2/5 pigs in this group died on 9th and 10th day after challenge, respectively.

### 3.2. Antibody Response

**Figure 3:**
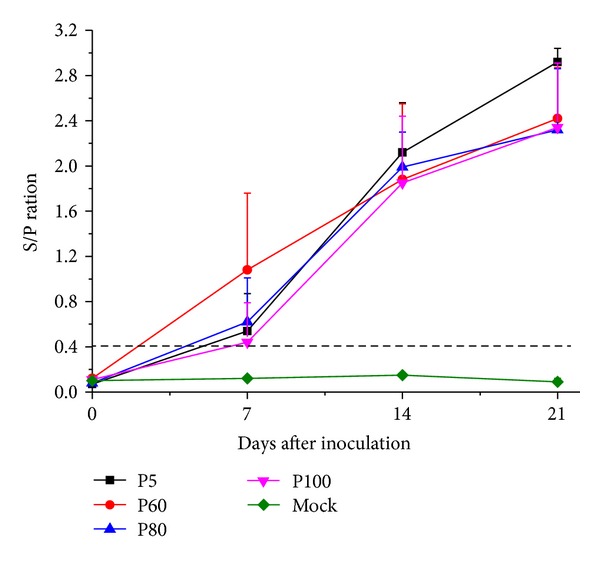
Mean anti-PRRSV antibody levels measured by ELISA. Samples were considered positive for antibody to PRRSV if the sample-to-positive (S/P) ratio was equal to or greater than 0.4. The geometric mean data with standard deviations (error bars) were shown.

**Figure 4:**
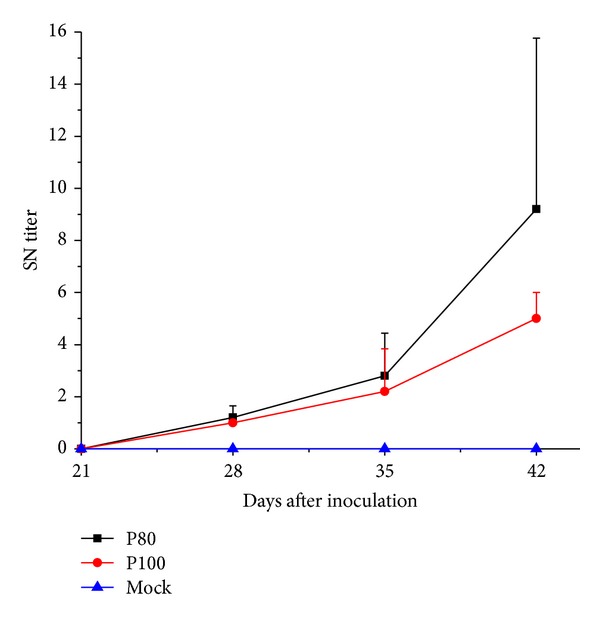
Virus neutralizing titers. Serum collected at −7, 0, 7, and 14 days after challenge were examined for neutralization with PRRSV. The data were expressed as the geometric mean ± S.D. of 5 pigs in each group. The serum was considered neutralizing at fourfold or higher dilution in positive response.

All pigs were negative for PRRSV antibody test at the time of inoculation (Figure 3). The serum of negative control pigs showed no responses for PRRSV antibodies throughout the course of the pathogenicity study. On 7 dpi, 7/10, 8/10, and 7/10 pigs were seroconverted in P5, P60, and P80 inoculation groups, respectively. P60 group showed a significant high S/P ratio (*P* < 0.05) than the other groups. 5/10 pigs in P100 inoculation group were seroconverted by 7 dpi. Consequently, all pigs were seroconverted to anti-PRRSV on 14 dpi. The virulent virus P5 inoculation group displayed a higher antibody titer compared to the other groups at 14 and 21 dpi (*P* < 0.05), respectively, while P60, P80, and P100 infectious group induced similar levels of anti-PRRSV antibody responses in pigs at these time points. Serum samples collected on 21, 28, 35, and 42 dpi were used for virus neutralizing assays. The results showed that PRRSV-specific neutralizing antibodies were absent before challenge and not detectable until 7th day after challenge. QY1 P80 group developed a better virus neutralizing reaction, demonstrating a higher level of serum neutralizing antibody against QY1 P5 at both days 35 and 42 than QY1 P100 inoculated group (Figure 4).

### 3.3. Viremia

**Figure 5:**
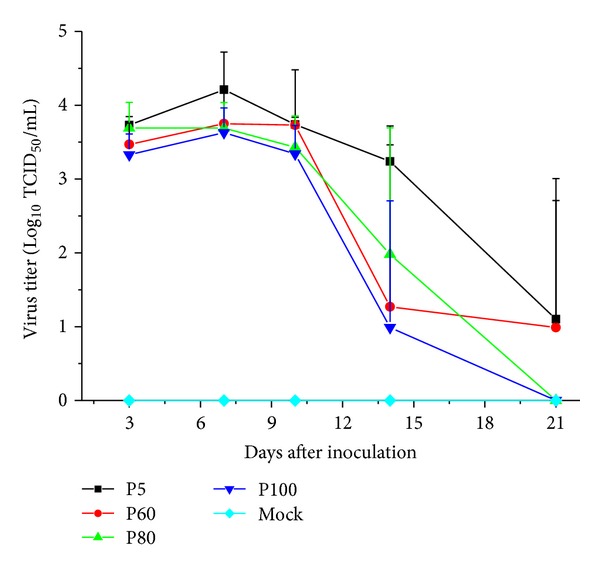
Level of viral titers on Marc-145 cells in the serum at each collection time point. The data were expressed as the geometric mean ± S.D. from 5 pigs in each group. No PRRSV was detected from any of the samples collected from pigs in the mock infected group.

Serum samples were analyzed by titration. The virulent virus P5 showed the highest viral titer at all collection time points, of which in the attenuation phenotype P100 was the lowest. After reaching the peak value on 7 dpi, titers of each group began to decline. There is less than one log decrease in growth before 14 days between the passage levels. One pig of QY1 P100 group maintained high levels of viremia whereas other pigs did not on 14 dpi, which caused a large variation at this time point (Figure 5). On 21 dpi, all pigs infected with P80 and P100 were PRRSV negative confirmed by both RT-PCR detection and viral isolation (Table 1).

### 3.4. Gross Lesions and Microscopic Lesions

**Figure 6:**
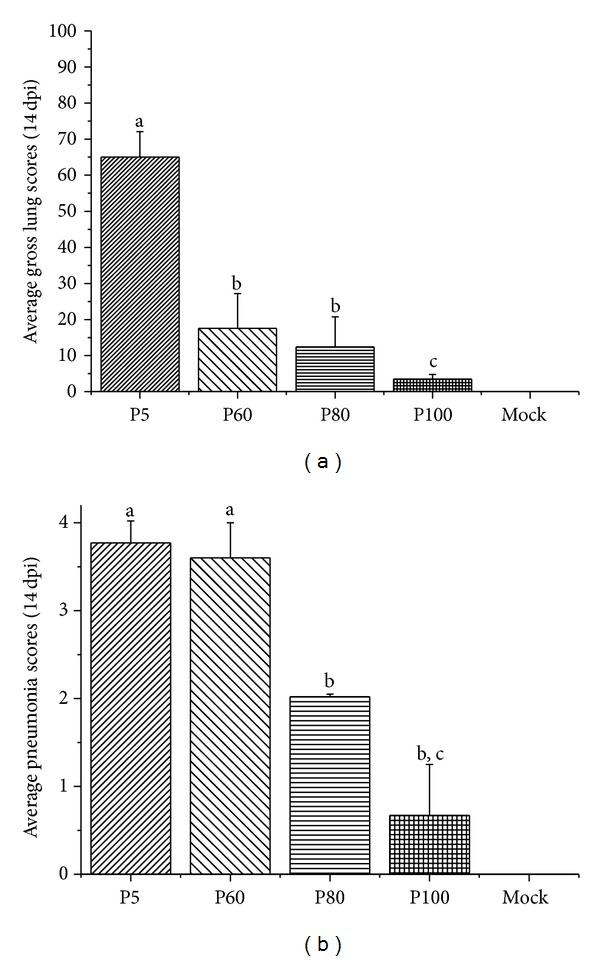
Average gross lung scores (a) and pneumonia scores (b) were recorded. The data were expressed as the mean ± S.D. from 5 pigs in each group. Different letter superscripts denote significant differences at *P* < 0.05.

**Figure 7:**
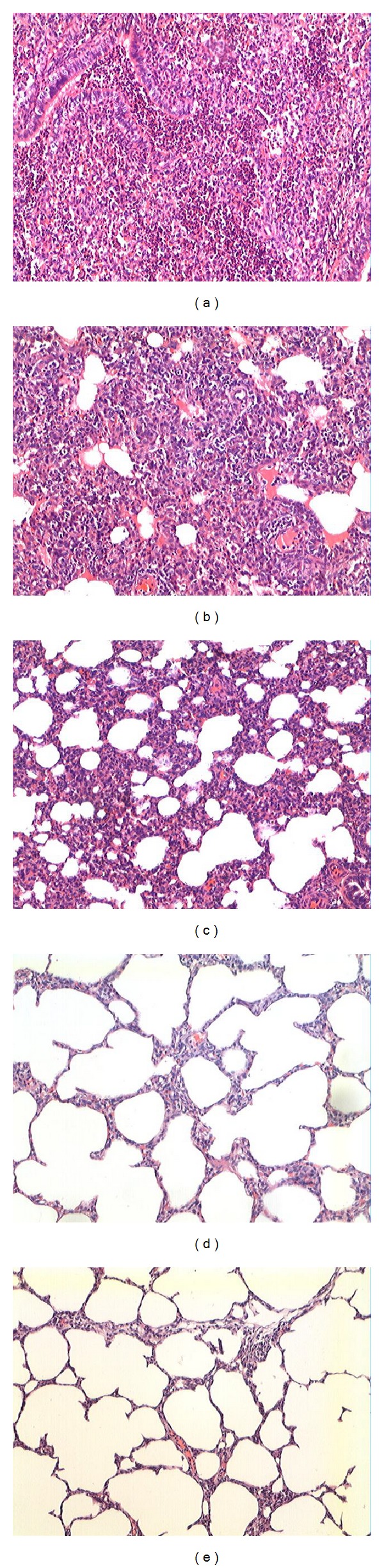
Histologic analysis of lungs from inoculated and control pigs. 14 days after inoculation, five pigs from three groups inoculated with P5 (a), P60 (b), P80 (c), and P100 (d) and the control group (e) were examined for histopathology.

Gross lung lesions scores were shown in Figure 6(a). At necropsy, pigs infected with PRRSV induce similar lesions but different in severity among groups. All pigs inoculated with virulent P5 virus showed obvious lung lesions, which affected the cranial, middle, and accessory, characterized by the lung's failure to collapse and the parenchyma being firm and rubbery. Furthermore, the average lung lesion score in this group was significantly higher (*P* < 0.01) than other PRRSV infected groups. 2/5 pigs in P60 inoculated group displayed moderate lesions and the other pigs in this group developed mild lung lesions. Mild lesions were also observed in P80 inoculated group, whose average lung lesions were similar for all the five pigs in this group. Gross lung lesions above 10% in P60 and P80 groups indicated residual pathogenicity in these passage levels. No obvious lung lesions were observed in the P100 and negative control groups. The varying degree of severity of microscopic lung lesions among each group was consistent with the display of gross lung lesions. Virulent P5 virus infected groups developed acute PRRSV infection signs, which were characterized by collapsed alveoli with an infiltration of macrophages and an accumulation of immature lymphocytes in the interstitium (Figure 7). Scores of microscopic lung lesions of pig inoculated with virulent P5 virus were significantly higher (*P* < 0.01) than that of P80, P100, and mock groups (Figure 6(b)). Lung lesions in P60 and P80 infected group were moderate on 14 dpi while P100 inoculated group was minimal or absent at this time point. Pigs inoculated with P80 or P100 virus were sacrificial on 14 day post-challenge no obvious pathological damage was found, indicating that pigs could tolerate lethal challenge.

### 3.5. Sequence Analysis

The full-length nucleotide sequences of QY1 P5 strain and its derivative passages P60, P80, and P100 were determined. The sequence data showed that the genomic sequence of QY1 P5 was 15,357 nucleotides (nts) in length, including a 189-nt 5′UTR, a 14,981-nt coding region comprising 10 ORFs, and a 187-nt 3′UTR with 37-nt ploy (A) tail. Comparative genomics analysis revealed that QY1 P5 shared a high identity with the HP-PRRSV strains including JXA1, HuN4, SY0608, and WHU1, which was 98.6%–99.6% homology at the nucleotide level. The unique molecular feature, discontinuous 30-amino acid deletion in Nsp2, was observed which was known to be one of the characteristics of the epidemic strains isolated in China in 2006 [2, 14]. The QY1 P5 showed 89.6% nucleotide identity with VR-2332 strain, but only 61.3% sequence identity with LV strain, revealing that QY1 P5 belongs to the HP-PRRSV. Sequence identities between the parental strain P5 and its three derivatives (P60, P80, and P100) were ranging from 99.6% to 99.8% at the nucleotide level. Compared with the parental strain, the numbers of nucleotide substitutions that occurred in P60, P80, and P100 were 29, 40, and 48, respectively. Taken together, all these implied that virus mutations increased over time, although some of these mutations were synonymous or were unable to pass from one generation to the next. In addition, two deletions appeared during the in vitro passage, with one nucleotide acid deletion occurring in the 5′UTR, besides the other novel noncontinuous 34-amino acid deletion located in nsp2 between positions 464 and 498. No insertion was observed during the series of passages. The RT-PCR and sequence analysis of viral RNA isolated from serum sample (*n* = 5) collected from P100 inoculated group 14 days after challenge confirmed the unique deletions were genetically stable in in vivo study.

### 3.6. Amino Acid Mutations during the Attenuation Process

Compared to the QY1 parental strain P5, a total of 47 altered nucleotides resulting 34 amino acid mutations were found in QY1 P100 genome which were located in ORF1a, ORF1b, ORF2a, ORF2b, ORF3, ORF4, and ORF5. Among the 34 amino acid mutations, 20 of 34 occurred in nsps and 14 mutations were found in the structural proteins. No mutation was found in the M and N protein-encoding region. Compared to the genome sequence of QY1 P5, P60 with 11 amino acid mutations was observed in P60, while 14 mutations occurred from P60 to P80 and 8 mutations were found from P81 to P100. Detailed analysis of the nucleotide mutations and corresponding amino acid changes was shown in Table 2. Mutations that occurred in the viral attenuation process would somehow be associated with viral attenuation. Compared to the other test virus, we proposed that the following virulence-associated amino acids were related to the PRRSV attenuation (Table 3). Additionally, a unique 34 aa deletion (464–498, corresponding to VR-2332) was identified in the nsp2. Compared to the deletion location found in TJM, a commercial attenuated-live vaccine strain with continuous deletion of 120 aa (628–747, corresponding to VR-2332) in the nsp2 protein [24], the deletion found in the QY1 P100 located upstream of nsp2.

## 4. Discussion

Mutations associated with PRRSV attenuation had been revealed in several studies; the investigators suggested that multiplate regions would relate to the attenuation of PRRSV [18, 24, 30–33]. In this study, a highly pathogenic PRRSV strain named QY1 P5 was passaged in vitro, and mutations occurred during this process, including 34 amino acid changes, 1 nucleotide deletion, and 34 amino acid deletions in the QY1 P100. Mutations were found in the genome except for nsp4, nsp6, nsp12, ORF6, and ORF7. Animal experiment revealed that QY1 P5 had been gradually attenuated in vitro. Pigs inoculated with QY1 P100 did not show any clinical signs, while signs of fever were observed in P80 infection pigs (4/10) during 5 to 10 dpi. Pigs inoculated with P60 showed mild-to-moderate clinical signs and QY1 P5 infected pigs developed acute HP-PRRSV infection symptoms, including high fever and severe respiratory disease. The virus attenuation was further supported by pathological examination, which revealed that pigs inoculated with the latter passage virus exhibited attenuated phenotype compared to animals inoculated with early passage virus.

The QY1 P5 strain had the typical genomic character of the HP-PRRSV strains and exhibited 98.6%–99.6% nucleotide identity with the Chinese HP-PRRSV strains. The present study clearly demonstrated that PRRSV strain QY1 P5 is a highly pathogenic virus. QY1 P5 caused typical clinical signs of “swine high-fever syndrome”; two pigs inoculated with P5 died. Animals infected with QY1 P5 reach over 41°C on 6 dpi and lasted for 5 days, and the virus replicated in swine to a high virus level in serum. Conversely, no pigs infected with the other intermediate viruses reach a temperature of 40.5°C and lower virus level in serum was observed in these groups compared to the P5 infected group. These results indicated that virulence of the QY1 P5 strain was attenuated remarkably after serial in vitro passage.

The 5′ and 3′UTR of PRRSV were shown to be important in the viral replication and transcription of PRRSV [34]. Studies of poliovirus vaccine identified mutations in the 5′-leader as important determinants for the attenuation of the vaccines [35]. However, no studies had showed that the mutations found in the 5′UTR were associated with PRRSV attenuation. 5′UTR was highly conserved during the PRRSV attenuating process [30]. Compared to VR-2332 strain, most of the HP-PRRSV isolates exhibited only one nucleotide deletion at position 119 within 5′UTR. In the present study, there was an additional guanine deletion that occurred at position 120 of 5′UTR during the QY1 attenuation process (at the early passage of 19); however, viral derivatives QY1 P60 which contained the continuous two nucleotide deletions in 5′UTR still showed mild-to-moderate virulence. Additionally, similar deletion had also been found in field strains BJPG and GX1002 with unknown virulence. The deletions found in 5′UTR were interesting; however, the relative contribution of such deletion to the attenuation of QY1 remains undefined and needs to be addressed in future studies.

Nsp2 gene deletions in both PRRSV genotypes were naturally found in the field [27, 36–38]. It had been demonstrated that as many as 403 amino acids within the nsp2 hypervariable region were dispensable for viral viability in vitro [39] and the selected nsp2 gene deletion mutations could delay seroconversion and growth attenuation in swine [40]. In this study, a unique 34 aa deletion (464–498, corresponding to VR-2332) was identified in this region. As a result, it was showed that the mutants with the new deletion in nsp2 showed higher titers than the original virus when cultured in Marc-145 cells (about 2 log10 of TCID50/mL). However, both mutants QY1 P80 and P100 growth attenuated in pigs and exhibited lower level of viral replication in vivo than their parental strains. It was known that a continuous deletion of 120 aa (628–747, corresponding to VR-2332) in the nsp2 protein was found in a commercial attenuated-live vaccine strain TJM [24]. Compared to the deletion location found in TJM, the deletion found in the QY1 P100 located upstream of nsp2 and such deletion found in QY1 P100 was similar to a HP-PRRSV isolate GDQY2 that we reported previously, which possesses a 35 aa deletion at amino acid positions 470–505 corresponding to VR-2332 [27]. Large spontaneous deletion in the nsp2 gene during in vitro cell culture passage has been reported previously, but such a deletion was found to have no effect on PRRSV virulence [41]. Thus, deletions found in the nsp2 of QY1 P100 were probably not the major factor for PRRSV virulence decrease. However, as the HP-PRRSV with a unique discontinuous deletion of 30 amino acids was the dominant strain of PRRSV in mainland China, the attenuation phenotypes of QY1 P100 with a new deletion in nsp2 would serve as a satisfied marker attenuated live-vaccine candidate.

Serial passage is a method that has been commonly applied for PRRSV MLV vaccines development, yet the mechanisms involved in PRRSV attenuation are largely unknown. It had been shown that major virulence determinants are located in NSP3-8 and ORF5, while the other NSPs and ORF2 were also involved in virulence determinants [42]. Based on our findings, the results revealed that determination sites of viral attenuation were variable and multiple, and incidental mutations always occurred in the nonstructural gene while the structural gene, especially the envelope-associated protein encoding gene, had unique amino acids changes among the attenuated PRRSV strains. G^12012^  →  A^12012^ change resulted in a synonymous mutation at L^10^ located within the predicted signal sequence of GP2, which was detected during sequential passages of DGQY1, and this nucleotide mutation also altered the amino acids mutation D^9^  →  N^9^ in E protein. Since parallel mutation in this position was found in other PRRSV viral attenuating process, it is possible that mutation achieved at this position was responsible for the viral attenuation. Another residue change (L^48^  →  F^48^) located in ORF2 was also found in two commercial MLV vaccines JXA1R and HUN4-F112, which seemed to be a unique molecular signature among the HP-PRRSV attenuated strains. It was shown that GP3, as a structural protein [43] presenting on virion envelope, was potentially associated with viral attenuation [30]. In this study, mutations (aa, H^79^  →  Y^79^, F^143^  →  L^143^) located in the antigenic region of GP3 were detected, and this mutation could influence the characteristics of antigenic sites of GP3. Besides, a varying mutation amino acid position (D^43^  →  N^43^) that was found in GP4 led to an additional potential glycosylation. Thus, GP4 of QY1-P100 was likely to have five N-glycosylation sites at positions N37, N43, N84, N120, and N130, respectively. GP4 was the key glycoprotein of PRRSV that was responsible for formation of the multiprotein complex, and the amount of glycan moieties could affect the viral attachment with cell receptor CD163 [44]. However, whether N43 glycan moieties could promote the viral propagation on Marc-145 cell or affect the virulence needs further investigations. An interesting observation was a mutation at position 196 of ORF5 (aa Q^196^  →  R^196^), and this change appeared in GP5 of HuN4 and TJ attenuation phenotypes as well. As was reported by Tian, the GP5Q^196^WGRL/P^200^ epitope was immunodominant and highly conservative in all type II PRRSVs and the 53D8 MAb could not recognize this mutated R^196^WGRL/P^200^ by IFA or IPMA [19].

The application of attenuated live vaccine such as RespPRRS MLV, JXA1-R, HuN4-F112, and TJM had greatly reduced the production loss caused by PRRS in China [3, 20, 30]. These live attenuated vaccines had been developed by serial passage on cell culture. In our study, QY1 P100 conferred very mild reactogenicity and high degree of immunogenicity, and pigs could tolerate lethal challenge of virulent virus, indicating that QY1 P100 was safe and satisfactorily immunogenic against HP-PRRSV. However, the protection of QY1 P100 under challenge by heterologous PRRSV strains was undefined. Such questions will be addressed experimentally in the future.

In conclusion, the data obtained from this study suggested that HP-PRRSV isolate QY1 was gradually attenuated during the in vitro passage. The virulence of PRRSV was determined by multiple factors in both structural and nonstructural genes. The results presented in the study signified some important clues for the attenuated PRRSV phenotype. The attenuated PRRSV phenotype QY1 P100 was proved to be immunogenic and protective in pigs, which could serve as a desired candidate for the attenuated live vaccine against HP-PRRSV under further evaluation.

## Figures and Tables

**Table 1:** Development of viremia in inoculation pigs.

Groups	Days after inoculation						Days after challenge		
	0	3	7	10	14	21	0	7	14
P5	0/10	10/10	10/10	10/10	7/10	3/5	—	—	—
P60	0/10	10/10	10/10	10/10	6/10	3/5	—	—	—
P80	0/10	10/10	10/10	10/10	8/10	0/5	0/5	0/5	0/5
P100	0/10	6/10	7/10	6/10	4/10	0/5	0/5	1/5	0/5
MOCK	0/10	0/10	0/10	0/10	0/10	0/5	0/5	5/5	5/5

**Table 2:** Nucleotide and amino acids changes among P5, 60, 80, and 100 of PRRSV strain GDQY1.

ORFs	Encoding protein (aa length^a^)	nt position^b^	P5	P60	P80	P100	aa position^c^	P5	P60	P80	P100
ORF1a	Nsp1*α* (180)	333	T	C	C	C	48	G	G	G	G
		677	A	C	C	C	163	H	P	P	P
	Nsp1*β* (203)	986	C	C	T	T	266	P	P	L	L
	Nsp2 (950)	1586	A	G	A	G	466	D	G	D	G
		1813	A	A	G	G	542	K	K	E	E
		2000	A	A	A	G	604	Q	Q	Q	R
		2018	T	T	T	G	610	I	I	I	T
		2527	A	A	G	G	780	K	K	E	E
		2560	A	A	A	G	791	M	M	M	V
		2729–2830			—^d^	—	846–880			—	—
		2986	A	G	G	G	933	E	G	G	G
		3178	G	A	A	A	997	D	N	N	N
		4220	G	G	A	A	1344	R	R	K	K
	Nsp3 (446)	4447	C	C	C	T	1419	C	C	C	C
		5120	C	C	C	T	1644	A	A	A	V
	Nsp5 (170)	6076	A	G	G	G	1963	E	K	K	K
		6156	C	T	T	T	1989	V	V	V	V
		6157	A	C	C	C	1990	K	Q	Q	Q
		6177	T	C	C	C	1996	F	F	F	F
		6248	G	G	A	A	2053	L	L	L	L
		6357	C	T	T	T	2056	A	A	A	A
	Nsp7 (259)	7134	A	A	T	T	2315	K	K	N	N
ORF1b	Nsp9 (643)	8139	G	G	G	T	178	A	A	A	S
		8310	G	G	A	A	235	E	E	K	K
		8322	T	T	A	A	239	L	L	I	I
		8427	C	T	T	T	274	P	S	S	S
		8269	A	A	G	G	554	I	I	M	M
	Nsp10 (441)	10895	C	C	T	T	408	F	F	F	F
	Nsp11 (223)	11321	G	G	G	A	109	K	K	K	K
OR2a	GP2 (257)	12012	G	A	A	A	10	L	L	L	L
		12131	A	A	T	T	50	Y	Y	F	F
		12334	A	A	G	G	118	I	I	V	V
		12377	G	G	A	A	132	S	S	N	N
		12592	C	C	A	A	204	H	H	N	N
ORF2b	E (73)	12012	G	A	A	A	9	D	N	N	N
		12131	A	A	T	T	48	L	L	F	F
ORF3	GP3 (255)	12840	C	T	T	T	79	H	Y	Y	Y
		13032	T	C	C	C	143	F	L	L	L
		13275	T	T	T	C	223	H	H	H	H
		13277	G	G	A	A	224	Q	Q	Q	Q
ORF4	GP4 (179)	13275	T	T	T	C	42	S	S	S	P
		13277	G	G	A	A	43	D	D	N	N
		13561	C	C	T	T	137	H	H	H	H
ORF5	GP5 (200)	13853	G	G	G	T	52	G	G	G	V
		13864	T	T	T	C	56	L	L	L	P
		13936	G	T	G	T	80	G	V	G	V
		14033	C	T	T	T	112	Y	Y	Y	Y
		14284	A	G	G	G	196	Q	R	R	R

^a^Amino acids length of each NSP.

^b^Nucleotide position mapped in the viral genome.

^c^Amino acids position mapped in each nonstructural or structural protein.

^d^Dash stands for deletions in those positions.

**Table 3:** Nine unique amino acids positions that varied in PRRSV attenuation process.

VR-2332/RespPRRSV-MLV	JXA1/JXA1R	HuN4/HuN4-F112	TJ/TJM	QY1/QY100
GP2 (L10/F10)	GP2 (L10/F10)			GP2 (L10/F10)*
	GP2 (Y50/F50)	GP2 (Y50/S50)		GP2 (Y50/F50)
		GP2 (I118/V118)		GP2 (I118/V118)
E (D9/Y9)	E (D9/N9)	E (D9/N9)		E (D9/N9)
	E (L48/F48)	E (L48/F48)		E (L48/F48)
	GP3 (H79/N79)		GP3 (F143/L143)	GP3 (H79/N79)
	GP4 (D43/G43)	GP4 (D43/N43)	GP4 (D43/G43)	GP4 (D43/N43)
			GP5 (G80/V80)	GP5 (G80/V80)
		GP5 (Q196/R196)	GP5 (Q196/R196)	GP5 (Q196/R196)

*Synonymous mutation.
